# ACTN3 R577X polymorphism is not associated with team sport athletic status in Italians

**DOI:** 10.1186/s40798-015-0008-x

**Published:** 2015-03-27

**Authors:** Myosotis Massidda, Valeria Bachis, Laura Corrias, Francesco Piras, Marco Scorcu, Claudia Culigioni, Daniele Masala, Carla M Calò

**Affiliations:** 1 Department of Life and Environmental Sciences, University of Cagliari, SS 554, km 4.500, Monserrato, 09042 Italy; 2 FMSI CR Sardegna and Cagliari Calcio SpA, Viale Tiziano, Cagliari, 70-00196 Italy; 3 University of Cassino, Viale dell’Università, Cassino, 03043 Italy

## Abstract

**Background:**

The *ACTN3* gene may influence performance in team sports, in which sprint action and high-speed movements, regulated by the anaerobic energy system, are crucial to the ultimate success of a match. The aim of this study was to determine the association between the *ACTN3* R577X (rs1815739) polymorphism and elite team sport athletic status in Italian male athletes.

**Methods:**

We compared the genotype and allele frequency of the *ACTN3* R577X polymorphism between team sport athletes (*n* = 75), endurance athletes (*n* = 40), sprint/power athletes (*n* = 64), and non-athletic healthy controls (*n* = 192) from Italy. Genomic DNA was collected using a buccal swab. Extraction was performed according to the manufacturer’s directions provided with a commercially available kit (Qiagen S.r.l**.**, Milan, Italy).

**Results:**

Team sport athletes showed a lower frequency of the 577RR genotype compared to the 577XX genotype than sprint/power athletes (*p* = 0.044). However, the *ACTN3* R577X polymorphism was not associated with team sport athletic status compared to endurance athletes and non-athletic controls.

**Conclusions:**

Our results agree with a recent large-scale study involving athletes from Spain, Poland, and Russia. The *ACTN3* R577X polymorphism was not associated with team sport athletic status compared to endurance athletes and non-athletic controls.

## Key points

- The study showed that the *ACTN3* R577X polymorphism is not considered a candidate gene to influence the performance of team sport athletes, yet we confirmed that the *ACTN3* 577RR genotype is overrepresented in sprint/power athletes compared to those in team sports.
- As result, this study emphasizes the importance of considering well-defined groups of athletes with a clear reference phenotype in genetic association studies applied to elite sport performance, also suggesting possible inter-individual genetic differences between athletes in the same team sport.

## Background

Research in the field of genetics among elite athletic performers has focused primarily on the sport at the end point of the sports performance continuum (sprint/power vs. endurance performance), yet the type of sport in which both the aerobic and anaerobic energy systems are important for achieving a high level of performance, such as team sports or endurance sports, has received little attention.

One of the most common targets of investigation has been a single nucleotide polymorphism (SNP) in the *ACTN3* gene, which encodes the alpha-actinin-3 protein [1]. This polymorphism leads to a premature stop codon (X), rather than an arginine (R) at position 577. Alpha-actinin-3 deficiency (*ACTN3* 577XX genotype) occurs in approximately 16% of the global population [1] and is associated with variations in human muscle performance. The alpha-actinin-3 protein is almost exclusively expressed in fast, glycolytic, and type IIX fibers that are responsible for the generation of rapid forceful contractions during activities such as sprinting.

Meta-analyses [2,3] and a recent review [4] of the literature regarding the influence of *ACTN3* on athletic performance suggest that the *ACTN3* 577RR genotype is associated with sprint/power athletes among Caucasians. Recently, Mikami et al. [5] examined a large cohort of elite Japanese athletes (*n* = 299) and found that the *ACTN3* R577X genotype was associated with sprint/power performance among track and field competitors, especially running 100- to 200-m sprints, and that R allele was associated with relative peak power in male collegiate athletes [6]. On the contrary, a recent study of Caucasian and East Asian swimmers did not show an association between *ACTN3* polymorphism and elite swimmer status [7]. The *ACTN3* 577X allele and the 577XX genotype were significantly associated with only certain groups of elite endurance athletes [8-17].

Seto et al. [18] provided a mechanistic explanation for the effects of the *ACTN3* genotype on skeletal muscle performance in elite athletes and on adaptation to changing physical demands in the general population. The authors highlighted the emerging evidence of the roles of alpha-actinin-3 in the regulation of metabolic and signaling pathways, contractile properties, and muscle fiber type. They showed that alpha-actinin-3 deficiency resulted in increased calcineurin activity in mouse and human skeletal muscle and an enhanced adaptive response to endurance training. Only a few studies investigated the association between the *ACTN3* R577X polymorphism and team sport performance. Santiago et al. [19] examined the frequency of this *ACTN3* genotype in 60 top-level professional Spanish soccer players, showing that the percentage distributions of 577RR and 577RX genotypes (48.3% and 36.7%) were significantly higher and lower, respectively, than those in controls (*n* = 123; 28.5% and 53.7%) or endurance athletes (*n* = 52; 26.5% and 52%) (*p* = 0.041). In a previous study [20], we did not find a significant difference in top-level soccer players’ (*n* = 42) jump performance among the various genotypes.

Pimenta et al. [21] examined 200 Brazilian professional soccer players and found that 577RR carriers ran 10, 20, and 30 m faster than 577XX carriers. Furthermore, the former scored higher in jump tests compared to both 577RX and 577XX carriers.

Not all the studies, however, found an association between *ACTN3* R577X polymorphism and team sport performance [22-24]. Recently, Garatachea et al. [25] did not find an association between *ACTN3* genotypes and explosive leg power in elite basketball players or with the status of being this type of athlete. Finally, Eynon et al. [26] compared the genotype and allele frequencies of the *ACTN3* R577X polymorphisms in a relatively large cohort of team sport athletes (*n* = 205), endurance athletes (*n* = 305), sprint/power athletes (*n* = 378), and non-athletic controls (*n* = 568) from Europe and found that *ACTN3* R577X polymorphism was not associated with team sport athletic status. However, they did find that the 577RR genotype was overrepresented in sprint/power athletes compared to team sport athletes.

The aim of this study was to analyze the associations between *ACTN3* R577X polymorphisms and elite team sport athletic status in a group of Italian athletes and to compare their genotype distributions with elite sprint/power athletes, endurance athletes, and non-athletic controls.

## Methods

A total of 178 Italian male athletes (*n* = 74 team sport athletes, *n* = 64 sprint/power athletes, *n* = 40 endurance athletes) and 190 controls were recruited between 2004 and 2014. All participants were unrelated European men, and all had Caucasian lineage for at least three generations. According to individual best performances, the athletes were divided into two subgroups: ‘elite level’ (competitors in European/World Championships or in the Olympic Games) and ‘national level’ (competitors in national-level but not international-level events) (Table 1). Of the athletes, 101 (57%) were classified as elite level and the remaining 77 (43%) were classified as national level.

**Table 1:** **Genotype and allelic distribution and Hardy Weinberg equilibrium (HWE) among athletes and controls.**

	**Team sport**	**Power**	**Endurance**	**Control**
All (*n*)	74	64	40	190
XX	14%	6%	15%	12%
RX	54%	45%	55%	58%
RR	32%	49%	30%	30%
MAF	0.413	0.289	0.425	0.406
HWE *p* value	0.478	0.549	1.00	0.057
Elite (*n*)	28	43	15	
XX	14%	7%	13%	
RX	61%	35%	60%	
RR	25%	58%	27%	
MAF	0.446	0.250	0.433	
National (*n*)	46	21	25	
XX	15%	5%	20%	
RX	50%	67%	40%	
RR	35%	28%	40%	
MAF	0.394	0.364	0.400

(i) The team sport athletes (*n* = 74) included 64 soccer players and 10 hockey players. Of these, 8 hockey players competed at international level (World/European Championships), 20 soccer players competed at international level (including five World Championships), while 2 hockey players and 44 soccer players competed at the national level (Italian Professional Championships, ‘Serie A’).
(ii) The sprint/power athletes (*n* = 64) included 16 track and field sprinters, 1 sprint swimmer, 17 wrestlers, 11 power lifters, and 19 gymnasts. Of these, 11 sprinters, 1 swimmer, 4 wrestlers, 10 power lifters, and 17 gymnasts were in the elite-level group, while 5 sprinters, 13 wrestlers, 1 power lifter, and 2 gymnasts were in the national-level group.
(iii) The endurance athletes (*n* = 40) included 24 track and field long-distance runners and 16 triathletes. Of these, 15 triathletes were in the elite-level group while all runners and 1 triathlete was in the national-level group.

The genotype frequencies among these athletes were compared with those of the randomly selected healthy non-athletic Italian males (controls). The controls were selected if they did not participate regularly in any competitive or structured sport or physical activity.

All participants and their parent/guardian (for minors) provided written informed consent, and the study protocol and informed written consent procedure were approved by the Medical Ethics Committees of the Italian Sports Federations, in accordance with Declaration of Helsinki for Human Research of 1974 (last modified in 2000).

Genomic DNA was extracted from each athlete with a buccal swab during the years 2004 to 2014, according to the manufacturer’s directions provided with a commercially available kit (Qiagen S.r.l., Milan, Italy) [27]. Exon 16 of *ACTN3* was amplified through polymerase chain reaction using the following primers:

forward 5′-CTGTTGCCTGTGGTAAGTGGG-3′

reverse 5′-TGGTCACAGTATGCAGGAGGG-3′.

Polymerase chain reaction products were digested with DdeI enzyme. The 577R and 577X alleles (CGA and TGA codons, respectively) were distinguished by the presence (577X) or absence (577R) of a DdeI restriction site in exon 16. Allele 577X shows two fragments with 205 and 85 bp, while allele 557R presents three fragments with 108, 97, and 86 bp. The obtained fragments were separated by 10% polyacrylamide gel electrophoresis and stained with ethidium bromide [27]. To ensure proper internal control, for each genotype analysis, we used positive and negative controls from different DNA aliquots that were previously genotyped with the same method.

Chi-squared tests were used to test for the presence of Hardy-Weinberg equilibrium (HWE). Multinomial logistic regression analyses were conducted to assess the association between genotype and athletic status/competition level. In each case, analyses were made comparing 577XX (reference group) vs. 577RX, 577XX vs. 577RR (co-dominant effect), 577XX vs. 577RR and 577RX combined (dominant effect), and 577XX and 577RX combined (reference group) vs. 577RR (recessive effect). Significance was accepted when *p* ≤ 0.05. Chi-squared tests for HWE were conducted using Genepop (v. 9), while statistical analyses were conducted using STATISTICA (Statsoft v. 7)

## Results

Table 1 shows the genotype and allele frequency distributions in the non-athletes and athletes divided by group and subgroup. The genotype distributions met HWE (all *p* > 0.05) for all groups of participants.

Our data showed significant differences in genotype frequency between team sport athletes and sprinters (Table 2). Team sport athletes had a lower frequency of the 577RR genotype compared to the 577XX genotype than the sprint/power athletes (*p* = 0.044) (Table 2). In fact, the sprint/power athletes were approximately 1.5 times more likely to have the 577RR genotype (as opposed to the 577XX genotype) than team sport athletes. No association was observed for any of the genotypes with respect to the level of competition (elite vs. national level) (Table 3).

**Table 2:** **Odds ratios of genotypes for athletes and control groups according to sport type**

	**Team sports vs. control**	**Team sports vs. endurance**	**Team sports vs. sprint/power**
XX (ref)			
OR	1	1	1
RX			
OR	0.72	0.99	0.50
CI	0.32 to 1.63	0.23 to 4.18	0.14 to 1.73
*p*	0.520	1	0.384
RR			
OR	0.79	1.04	0.26
CI	0.33 to 1.89	0.21 to 4.98	0.07 to 0.95
*p*	0.654	1	*0.044*
RR and RX (XX ref)			
OR	0.75	1.01	0.38
CI	0.34 to 1.63	0.25 to 4.03	0.11 to 1.26
*p*	0.534	1	0.168
RR (RX and XX ref)			
OR	1.02	1.05	0.48
CI	0.57 to 1.83	0.35 to 3.08	0.23 to 0.96
*p*	1	1	0.053

OR, odds ratio; CI, confidence interval; *p*, two-tailed *p* value. Significance is assumed when *p* < 0.05. The value in italics is statistically significant.

**Table 3:** **Odds ratios of genotypes for elite athletes compared to national-level athletes in team sports**

**Genotype**	**Team sport**		
	**OR**	**CI**	***p***
XX (ref)			
RX	1.293	0.32 to 5.13	1
RR	0.765	0.16 to 3.48	1
RX-RR (XX ref)	1.076	0.28 to 4.07	1
RR (XX-RX ref)	0.625	0.21 to 1.78	0.444

CI, confidence interval; ref, reference; OR, odds ratio.

## Discussion

We tested, for the first time, the association between *ACTN3* R577X polymorphism and team sport athletic status in Italian athletes. Our results indicate that team sport athletes have a lower 577RR than 577XX genotype frequency, compared to sprint/power athletes (*p* = 0.044). No significant difference in the *ACTN3* R577X polymorphism genotype distribution was found between the team sport athletes, endurance athletes, and control group. Furthermore, the *ACTN3* R577X genotype distribution was similar between elite-level and national-level team sport athletes, suggesting that this polymorphism is not influential among team sport athletes.

Our findings agree with those of Eynon et al. [26], who recently analyzed elite team sport athletes from three European countries (i.e., Spain, Poland, and Russia) and showed that their performance was not significantly influenced by *ACTN3* R577X polymorphism. Similarly, our results agree with theirs in that team sport athletes were less likely to possess the 577RR genotype than the 577XX genotype compared to power athletes (*p* = 0.045).

The *ACTN3* R577X polymorphism was consistently associated with elite sprint/power status [2,3] and muscle power [6]. The functional consequence of the *ACTN3* R577X polymorphisms on sport performance is supported by the Actn3 knock-out (KO) mouse model [28]. The authors demonstrated that the loss of alpha-actinin expression results in a shift in muscle metabolism toward the more efficient aerobic pathway, with a subsequent increase in running distance achieved before exhaustion during a treadmill test [28]. Moreover, the Actn3 KO mice (*ACTN3* 577XX), compared to wild-type (WT) counterparts, had lower muscle mass (smaller diameter fast-twitch muscle fibers), significantly lesser grip strength, significantly lower anaerobic enzyme activity, and higher oxidative/mitochondrial enzyme activity without a shift in fiber-type distribution [29].

In team sports, most of the decisive actions during competition are characterized by jumping and/or sprinting, and it is for that reason that we hypothesized that the 577RR genotype would be associated with team sport performance. However, team sports generally involve mixed aerobic-anaerobic demands and players may not need to have extraordinary aerobic/anaerobic capacity. During a soccer game, for example, aerobic metabolism dominates energy delivery [30], yet the most decisive actions, characterized by jumping and sprinting, are supported by anaerobic metabolism. In similar team sports, many factors contribute to success, including muscle power, aerobic endurance, ball skills, and psychological traits. Therefore, muscle power represents just one of several factors that determine a player’s ability but a substantial development of this factor is decisive during the crucial phases of a match.

When studying the possible role of genetics in team sports, it should be noted that not all players have similar characteristics in terms of physiological demand because it varies based on the position played. For example, during a rugby match, players perform various activities based on their position [31]. Forwards, rather than backs, are involved in significantly more physical collisions and tackles, experience a greater proportion of high-intensity activity than low-intensity activity, and travel greater distances during a match (9,929 vs. 8458 m) [31], demonstrating that a wide range of skills and physiological demands exist for the different playing positions.

Indeed, while *ACTN3* R577X polymorphism has been previously associated with sprint/power or endurance performance, the results of this study suggest that it is not associated with team sport athletic status.

The most obvious limitation of this study is the sample size. However, the very large population samples required to reach a sufficient statistical power in the field of genetic studies do not harmonize with the limited number of elite athletes for each sport discipline. We believe that the small sample size could be justifiable by the number of top-level athletes and by the homogeneity of the sample in terms of geographical and ethnical origin, recruited for the present study.

## Conclusions

In conclusion, our results confirm, for the Italian population, the recent data published by Eynon et al. [26] that *ACTN3* R577X polymorphism is not significantly associated with team sport athletic status, when team sport athletes are compared to endurance athletes and non-athletic controls, and the 577RR genotype is overrepresented in power/sprint athletes compared to team sport athletes.
